# The Relationship between Compound Danshen Dripping Pills with Isosorbide Mononitrate in the Treatment of Elderly Patients with Unstable Angina Pectoris

**DOI:** 10.1155/2018/3429151

**Published:** 2018-07-18

**Authors:** Yulin Liang, Junbo Zou, Xiaofei Zhang, Yu Wang, Jia Tai, Dongyan Guo, Chunli Cui, Jing Wang, Jiangxue Cheng, Yajun Shi

**Affiliations:** ^1^Department of Pharmaceutics, College of Pharmacy, Shaanxi University of Chinese Medicine, Xianyang, Shaanxi, China; ^2^Department of Pharmaceutics, College of Pharmacy, The Key Laboratory of Basic and New Drug Research of Traditional Chinese Medicine, Shaanxi University of Chinese Medicine, Xianyang, Shaanxi, China

## Abstract

**Objective:**

To evaluate the clinical efficacy and safety of Compound Danshen Dripping Pill (CDDP) and Isosorbide Mononitrate (ISMN) in the treatment of unstable angina pectoris (UAP) in the elderly.

**Materials and Methods:**

CNKI, Wanfang, VIP, CBM, and PubMed databases were searched for appropriate articles without language limitations on keywords. RevMan 5.3 software was used to perform the meta-analysis.

**Results:**

This analysis compared CDDP with ISMN of 21 randomized controlled trials (RCTs) that involved a total of 2356 patients with UAP. When the treatment lasted for four weeks, the clinical effective rate was OR = 3.97, 95% CI = 2.97, 5.30, and* P* < 0.00001, the ECG efficiency was OR = 3.43, 95% CI = 2.13, 5.53, and* P* < 0.00001, and incidence of adverse reactions was OR = 0.73, 95% CI = 0.52, 1.04, and* P* = 0.08 > 0.05. When the treatment lasted for eight weeks, clinical efficiency rate was OR = 4.22, 95% CI = 2.37, 3.79, and* P* < 0.00001, incidence of adverse reactions was OR = 0.58, 95% CI = 0.26, 1.27, and* P* = 0.17 > 0.05, whole blood low-cut blood viscosity was SMD = -0.61 and 95% CI -1.60, 0.38, whole blood high-cut blood viscosity was SMD = -0.38 and 95% CI -0.97, 0.21, and blood cells specific volume was SMD = -0.80 and 95% CI -2.61, 1.01.

**Conclusion:**

Based on this meta-analysis, the CDDP was superior to ISMN with UAP in the elderly. However, there is still a need to further verify the clinical efficacy and safety of CDDP with more strictly designed RCTs with large sample and multiple centers in the future.

## 1. Introduction

UAP [1] is a common coronary syndrome between stable angina and acute myocardial infarction, which could lead to myocardial infarction or sudden death [2]; it is a clinically common cardiovascular disease (CVD) [3]. The pain can be induced; UAP is accompanied by accidental pains which would be induced even in the resting state [4]. It is similar to typical stable angina [5] but lasts longer. It is easy to evolve into acute myocardial infarction or sudden death, so timely diagnosis and correct treatment are needed urgently, including early onset angina pectoris, angina pectoris, spontaneous angina pectoris, X synthesis, supine angina pectoris, and postprandial angina pectoris. With the acceleration of population aging in China, the incidence of unstable angina pectoris is on the rise, threatening people's health and maybe threatening life if not treated promptly.

The CDDP is a new type of pure Chinese medicine drop that has been successfully developed based on the basic theory of traditional Chinese medicine and the use of modern medical technology [6]. Compared with the original dosage form, it has the advantages of smaller dosage, better curative effect, more prominent effect, fewer side effects, and reduced gastrointestinal irritation, and it is a commonly used traditional Chinese medicine preparation [7]. Its main components are* Salvia miltiorrhiza*,* Panax notoginseng*, and Dipterocarpaceae [8].* Salvia miltiorrhiza* [9] as a traditional medicine for promoting blood circulation and stasis has the effect of promoting blood circulation, relieving blood, and relieving pain and plays an important role in the treatment of cardiovascular diseases (CVD). The main component is water-soluble danshensu [10], which has the function of dilating blood vessels, increasing coronary flow, improving microcirculation, and so forth.* Notoginseng* saponins extracted from* Panax notoginseng* can increase coronary blood flow and reduce myocardial oxygen consumption and arterial pressure. Dipterocarpaceae has anti-myocardial infarction, reduced myocardial oxygen consumption, anti-inflammatory, and analgesic effects [11]. Therefore it has been widely used for treating cardiovascular diseases and peripheral circulation disorder clinically [2]. ISMN [12] is one of the most effective and frequently used agents for treating angina pectoris [13]. The main pharmacological effect is a relaxation of the vascular smooth muscle [14], which can effectively prevent the onset of angina [15].

In recent years, more and more RCTs on the CDDP and ISMN in the treatment of angina pectoris were compared [11, 16–18]. However, UAP was rarely considered; only a few studies have put emphasis on sample clinical measurements such as the clinical efficacy and the ECG efficiency. Since some drugs have different onset times and drug efficacy duration, different dose cycles may have different effects on efficacy. In this study, the meta-analysis was performed on the research data of different clinical dosing cycles to evaluate the clinical efficacy and safety of CDDP and ISMN in the treatment of elderly patients with UAP.

## 2. Materials and Methods

### 2.1. Inclusion and Exclusion Criteria

According to the suggestions of a cardiologist, we designed the inclusion criteria as follows: (1) We selected elderly patients who meet the diagnostic criteria for UAP. (2) The study was a randomized, double-blind controlled study. (3) The patients were between 30 and 90 years of age. (4) There was no limitation of race and gender of the study subjects. (5) Patients received general treatment, according to the different drugs to be divided into treatment group and control group; the treatment group used CDDP, and the control group used ISMN; in addition to this, two groups have no other treatment measures. (6) The duration of treatment is 4 weeks or 8 weeks. Exclusion criteria were (1) unclear diagnosis, (2) unmatched treatment cycles, and (3) unsatisfactory interventions

### 2.2. Retrieve Information

CNKI, Wanfang, VIP, CBM, PubMed and other databases were searched to retrieve information from RCTs of the CDDP and ISMN in the treatment of UAP in the elderly in recent years. Keywords were “compound Danshen dripping pills" and “unstable angina elderly" [Title/Abstract], “isosorbide mononitrate" and “unstable angina elderly" [Title/Abstract], “unstable angina elderly" [Title/Abstract], “compound Danshen dripping pills" [Title/Abstract], “isosorbide mononitrate" [Title/Abstract], and “unstable angina" [Title/Abstract]. RCTs were examined without language limitations in order to obtain a comprehensive retrieval published before 25 December 2017. All RCTs were screened according to certain criteria. And relevant RCTs were downloaded into Endnote software (version X8, Thomson Reuters, Inc., New York, USA) for further exploring. We have made detailed records and analysis of relevant data. Duplicate records were removed. The full-text review was performed, while the title/abstract was thought to be thematic.

### 2.3. Quality Assessment

This meta-analysis used Review Manager 5.3 software to perform quality assessment.

It was evaluated from random sequence generation, allocation concealment, blinding of participants and personnel, blinding of outcome assessment, incomplete outcome data, selective reporting, and other biases and divided into three indexes: “high risk,” “unclear risk,” and “low risk.”

### 2.4. Statistical Methods

Review Manager 5.3 (Cochrane Collaboration) statistical software was used for analysis and processing. Outcome measures such as the clinical efficacy, adverse reactions, and the ECG efficiency were regarded as dichotomous variables and presented as the odds ratio (OR) with 95% confidence intervals (95%). Blood viscosity was continuous variable that was presented as the Std. mean difference (SMD) with 95% CI. Q statistic and* I*^2^ tests were applied to assess the heterogeneity among studies. If* P* > 0.10 and* I*^2^ ≤ 50%, the study was homogeneous, using a fixed-effects model for statistical analysis. And a random-effects model was used to analyze data with heterogeneity (*P* ≤ 0. 10;* I*^2^ > 50%), and the effective results are statistically significant at* P* < 0.05 [11]. Potential publication bias was revealed by funnel plots.

## 3. Results

### 3.1. Literature Search

**Figure 1:**
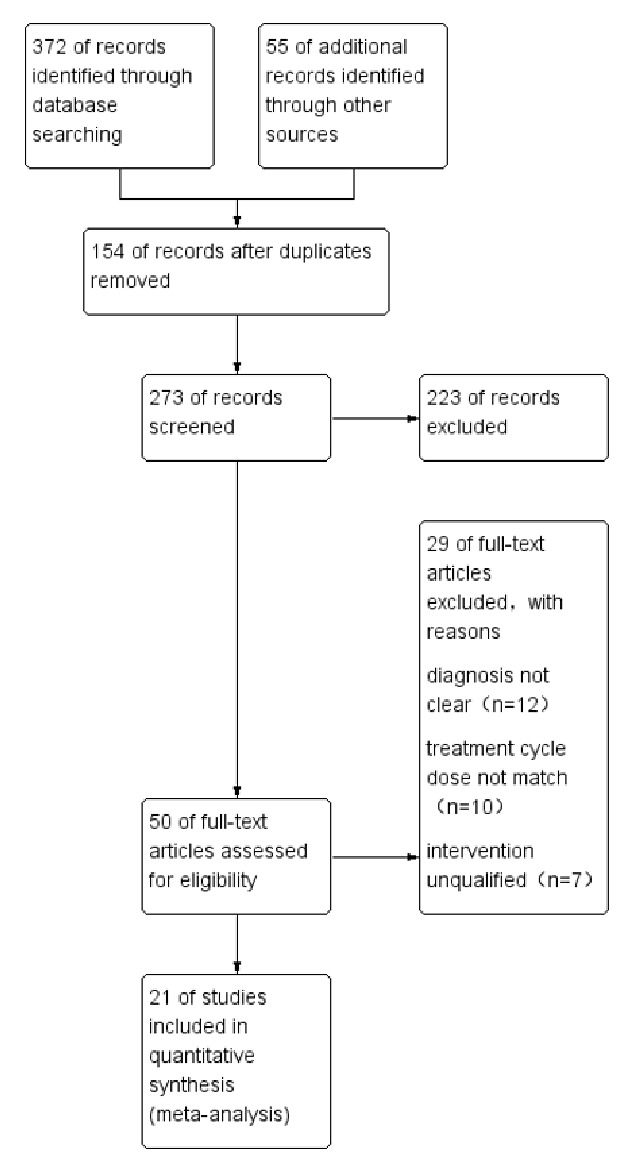
Studies selection process.

A total of 21 RCTs involving 2356 patients were included in this systematic review. Two people were retrieved and cross-checked when there are disagreements; the third person participates in the discussion. Studies selection process is shown in Figure 1.

### 3.2. Risk of Bias Assessment

According to the Cochrane risk of bias estimation, six RCTs referred to “random number table method" or similar methods [19–24]. Therefore, selection bias was evaluated as “low risk.” Although the remaining RCTs mentioned “random," they did not describe specific methods; selection bias was evaluated as “unclear risk.” Ten RCTs selection bias was remarked as “high risk” in allocation concealment [19, 21, 23, 25–31]. Three RCTs were remarked as “unclear risk” [20, 24, 32] and eight RCTs were “low risk” [22, 33–39]. Blinding of participants and outcome assessment of all RCTs were not mentioned, so performance bias and detection bias were deemed as “unclear risk.” There is no shortage of cases or selective reports, so the attrition bias and reporting bias were assessed as “low risk.” Although no other biases were found in these trials, considering their poor methodological quality, we decided to assign an “unclear risk” of bias to all the included trials.

### 3.3. Literature Screening

According to the above search terms, a total of 427 RCTs were consulted. Depending on the inclusion criteria and exclusion criteria, including unclear diagnosis, unmatched treatment cycles, and unsatisfactory interventions, only 21 RCTs match the standards. Patients in experimental group received CDDP therapy, whereas patients in control group received ISMN therapy only. Observed indicators include clinical effective rate, ECG changes, blood viscosity improvement, and adverse reactions. The detailed characteristics of the included 21 studies are shown in Tables 1 and 2.

### 3.4. Subgroup Analysis

In the 21 RCTs, the treatment cycle of 16 RCTs was four weeks, while that of 5 RCTs was eight weeks. Among them, after four weeks of treatment and the administration of the drug, besides the clinical curative effect, four articles reported the efficacy of electrocardiogram, eight articles reported the adverse reactions, and four articles reported the blood viscosity. After eight weeks of treatment, in addition to the clinical efficacy, only one article reported the efficacy of ECG [24] and two reported adverse reactions. Indicators will not be analyzed for they were mentioned in few studies.

#### 3.4.1. When the Treatment Lasted for Four Weeks

**Figure 2:**
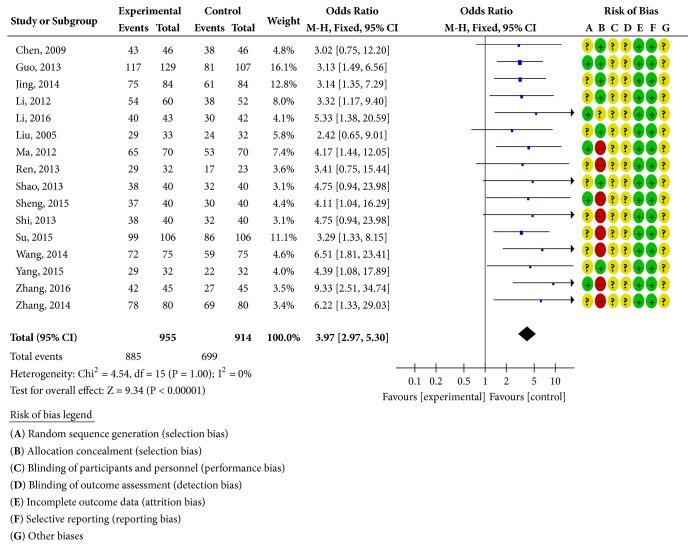
Clinical efficacy analysis chart.

**Figure 3:**
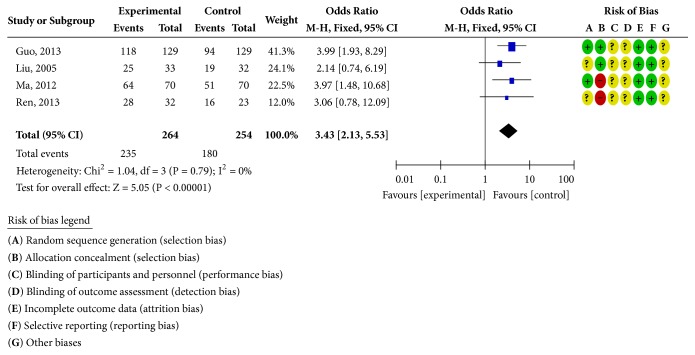
ECG efficiency analysis chart.

**Figure 4:**
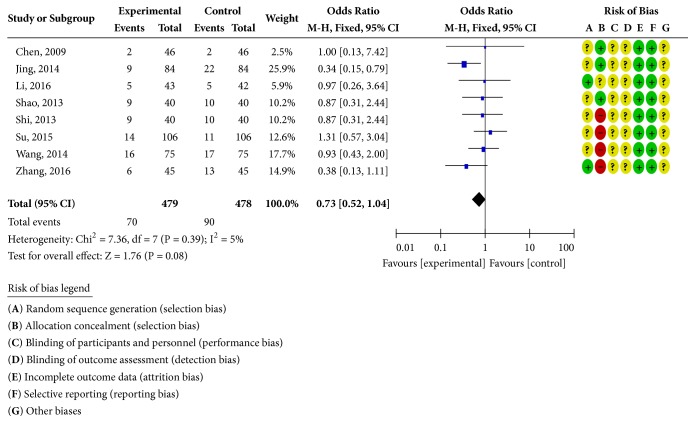
Adverse reaction rate analysis chart.

Sixteen RCTs reported clinical curative effects [19–23, 25–29, 34–39], as shown in Figure 2. There was homogeneity in each study (*P* = 1.00;* I*^2^ = 0%). Statistical analysis was performed using a fixed-effects model. The OR and 95% CI for clinically effective rate were OR = 3.97 and 95% CI = 2.97, 5.30) (*P <* 0.00001); the results showed that the clinical efficacy of the treatment group was better than the control group and the difference was statistically significant; four RCTs reported the ECG efficiency [22, 23, 25, 36], as shown in Figure 3. The study was homogeneous (*P* = 0.79;* I*^2^ = 0% ), using a fixed-effect model for statistical analysis; the OR and 95% CI for ECG efficiency were OR = 3.43 and 95% CI = 2.13, 5.53 (*P* < 0.00001); the results show that the ECG treatment group was more effective than the control group; the difference has statistical significance; eight RCTs reported adverse reactions [19, 20, 26–28, 35, 38, 39], as shown in Figure 4. There was homogeneity in each study (*P* = 0.39;* I*^2^ = 5%), using a fixed-effects model for statistical analysis; the OR and 95% CI for incidence of adverse reactions were OR = 0.73 and 95% CI = 0.52,1.04 (*P* = 0.08 > 0.05); the difference was not statistically significant and it may not be possible to draw a definite conclusion that the incidence of adverse reactions is lower than ISMN, due to the sample size and treatment cycle, and this remains to be further explored.

**Figure 5:**
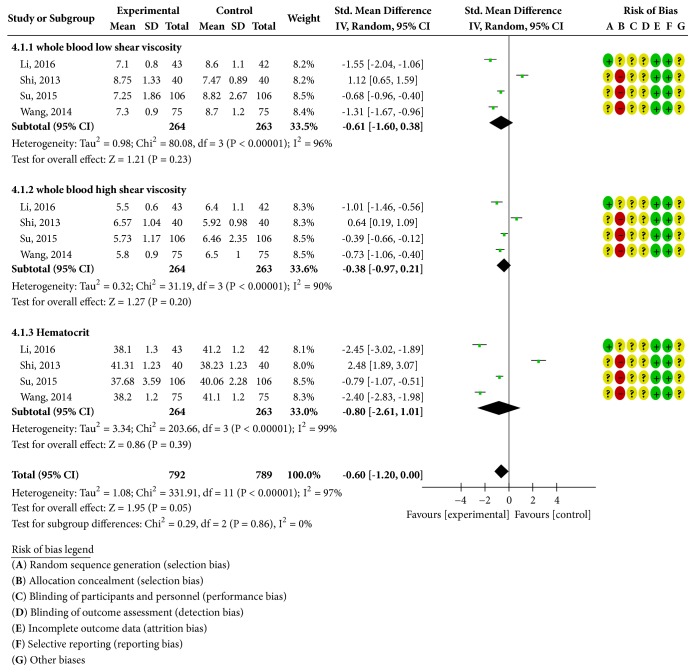
Blood viscosity change analysis chart.

Four RCTs reported whole blood low-cut blood viscosity [20, 26–28], as shown in Figure 5. There was heterogeneity in each study (*P* < 0.00001;* I*^2^ = 96%); a random-effects model was used for statistical analysis; the statistical analysis of the model showed that the improvement of the low-cut viscosity of the whole blood in the experimental group was better than that of the control group. The difference was statistically significant [SMD = -0.61; 95% CI = -1.60, 0.38], and four literature studies reported that the whole blood high-cut blood viscosity was heterogeneous in each study (*P* < 0.00001;* I*^2^ = 90%); a random-effects model was used for statistical analysis; the improvement of whole blood hyperviscosity in the experimental group was better than that in the control group. The difference was statistically significant [SMD = -0.38; 95% CI = -0.97, 0.21]. Hematocrit was reported in four studies (*P* < 0.00001;* I*^2^ = 99%); a random-effects model was used for statistical analysis; the results showed that the hematocrit of the experimental group improved better than the control group. The difference was statistically significant: [SMD = -0.80; 95% CI = -2.61, 1.01].

#### 3.4.2. When the Treatment Lasted for Eight Weeks

**Figure 6:**
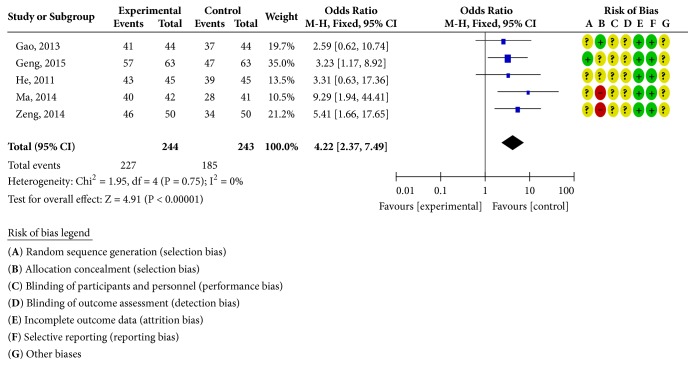
Clinical efficacy analysis chart.

**Figure 7:**
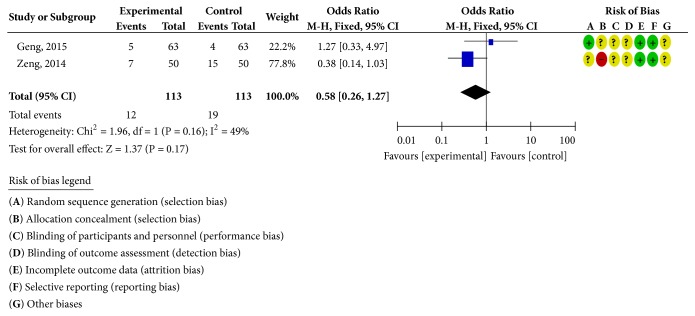
Adverse reaction rate analysis chart.

Five RCTs reported clinical outcomes [24, 30–33], as shown in Figure 6. There was homogeneity in each study (*P* = 0.75;* I*^2^ = 0%). Statistical analysis was performed using a fixed-effects model; the OR and 95% CI for clinically effective rate were OR = 4.22 and 95% CI = 2.37, 3.79 (*P* < 0.00001); the results showed that the clinical efficacy of the treatment group was better than the control group; the difference was statistically significant; two RCTs reported adverse reactions [24, 31], as shown in Figure 7. There was homogeneity in the studies (*P* = 0.16;* I*^2^ = 49%). Statistical analysis was performed using a fixed-effects model; the OR and 95% CI for the incidence of adverse reactions were OR = 0.58 and 95% CI = 0.26, 1.27 (*P* = 0.17 > 0.05). There was no statistically significant difference. It may not be possible to draw a definite conclusion that the incidence of adverse reactions is lower than ISMN, due to the sample size and the treatment cycle, and this remains to be further explored.

### 3.5. Publication Bias

**Figure 8:**
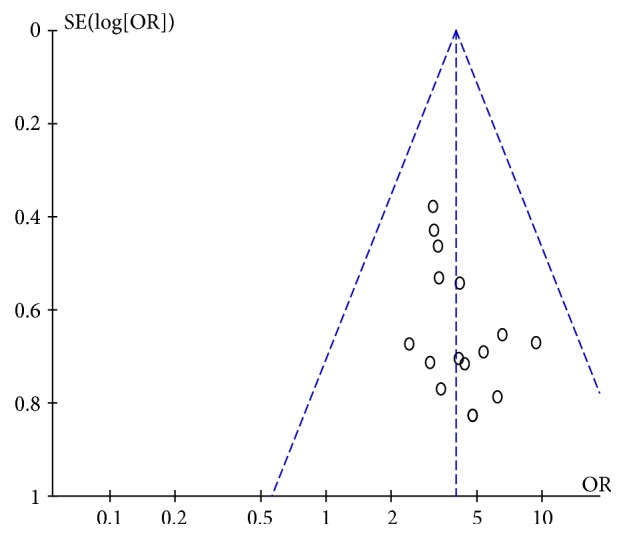
Publication bias analysis chart (4 weeks).

**Figure 9:**
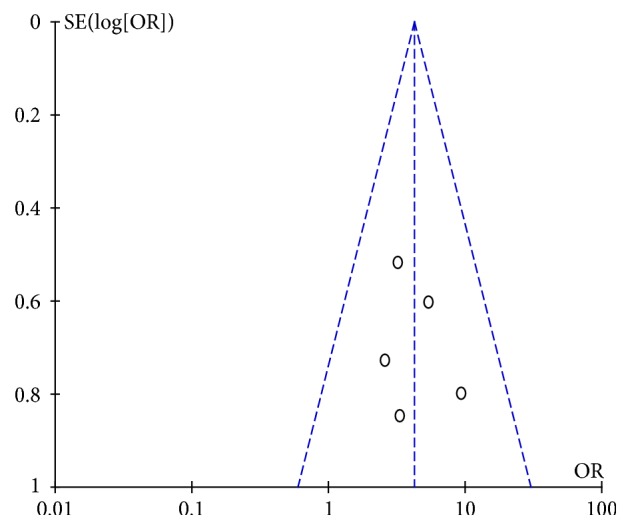
Publication bias analysis chart (8 weeks).

Funnel chart analysis of clinical efficacy found the distribution of scattered points of symmetry, indicating that the possibility of publication bias is small, as shown in Figures 8 and 9.

## 4. Discussion

Elderly UAP [40] is a common clinical cardiovascular disease (CVD); with the growing incidence of aging in China, the incidence of UAP in elderly patients increased year by year (nearly 300 million patients with CVD in China); increasing of the affected population has brought about huge harm to people's health. According to the survey, two out of every five deaths are cardiovascular patients in China, and CVD has become the first killer [41]. The main pathogenesis is coronary atherosclerotic plaque instability, easy to cause coronary artery obstruction and spasm after rupture, making local myocardial ischemia and hypoxia; if the treatment is not timely, the patients are likely to be attacked by acute myocardial infarction. In the theory of traditional Chinese medicine, the UAP is defined as “thoracic obstruction” and “precordial pain with cold limbs” which is involved with inward invasion of pathogenic cold, endogenous impairment due to overstrain, body deficiency due to old age, blood stasis due to cold, and Qi stagnation. ISMN is commonly used clinically as traditional antiangina drugs; its main pharmacological effects are to relax the vascular smooth muscle, dilate coronary artery, increase coronary blood flow, reduce myocardial oxygen consumption, reduce cardiac load, delay myocardial remodeling, and effectively relieve angina in patients [42].

Chinese patent medicine plays an important part in the prevention, treatment, and first aid of CVD. The CDDP, also known as the “Dantonic Pill,” is a representative Chinese patent drug with the function of activating blood circulation and removing blood stasis [43]. Its main components are* Salvia miltiorrhiza*,* Panax notoginseng* extract, and Dipterocarpaceae, which were highly dispersed in excipients such as polyethylene glycol 2000 (PEG-2000) and PEG-4000. Since it was listed in 1994, the CDDP has accumulated more than 450 million person-times and has accumulated a wealth of valuable clinical experience. Benefiting from the highly dispersed state, the active ingredients are more easily absorbed through the intestinal mucosa; then the bioavailability is enhanced significantly. Meanwhile, disadvantages of traditional tablets such as stomach injury and mucosa irritation are overcome as well [44]. Modern pharmacology studies have confirmed CDDP with coronary artery expansion, protection of vascular endothelial cells, antiplatelet aggregation, antithrombosis, improvement of microcirculation and other effects, the CDDP chemical composition including water-soluble danshensu [10], salvianolic acid B [45], protocatechuic aldehyde [46], and so on. Water-soluble danshensu can reduce platelet aggregation with anticoagulant, lipid-lowering antagonism of calcium and inhibit fibroblast proliferation and secretion of the matrix and also acts as an anti-inflammatory agent by inhibiting the adhesion molecules on the cell surface. The main component of* Panax notoginseng* is total* Panax notoginseng* saponins [47], and Dipterocarpaceae is a dispersion in the form of a solid, produced by a special process. These chemical components have the characteristics of fast dissolution, uniform dispersion, and high purity. The CDDP can also directly act on mucosal cells of patients to reduce the drug's timeliness, increase the bioavailability, and reduce stomach discomfort. Chinese medicine combination can play an antioxidant role and effectively inhibit the activation of hepatic stellate cells, thus reducing the necrosis of liver cells. In addition, its bioavailability is high; it reduces and relieves neuropeptide dysfunction caused by hypoxia after traumatic brain injury and clears the blood stasis; it is important for antithrombotic formation and anticoagulation and for reducing epileptiform discharge; it improves plasma NO concentration, reduces brain damage and brain edema, promotes brain tissue repair, and improves convulsive threshold [7]. The safety of CDDP has been demonstrated by several experiments about acute toxicity, long-term toxicity, teratogenesis, and carcinogenesis [48].

Meta-analysis is a method that uses statistical methods to analyze and summarize the numerous research data that were collected. It is essentially an observational study, but it also follows the basic principles of scientific research, including related papers search, literature inclusion and exclusion criteria, extracting data information, statistical processing, reporting results, and other basic research processes. Compared with general analytical studies, meta-analysis processes the published data instead of analyzing the raw data of each observed object in the independent study [49].

In this study, a total of 21 RCTs were screened out for meta-analysis; the data were divided into treatment cycles of four weeks and eight weeks, with ISMN as a control group and the CDDP as a treatment group to evaluate the clinical efficacy. The meta-analysis' results confirmed that the clinical efficacy and ECG efficiency of the two groups were significantly different (*P* < 0.05), indicating that the CDDP is better than ISMN. In terms of safety, we found that few RCTs reported relevant adverse reactions, headaches, dizziness, and facial flushing symptoms that may occur in patients; the types of adverse reactions were systemic reactions, skin and accessory reactions, and nervous system reactions [2]. However, due to the small sample size and the treatment cycle, we cannot yet draw a clear conclusion, and this remains to be further explored.

The distribution of the clinical efficacy samples around the funnel was even and symmetrical, indicating that there was a low possibility of publication bias. However, eight weeks of clinical efficacy samples were unevenly distributed, mostly concentrated on the left side, and the symmetry was not very strong. Therefore, it is more likely to be biased, but it may also be due to the small number of samples.

## 5. Conclusion

In summary, according to the comparison of clinical efficacy, ECG efficiency, blood viscosity, and other indicators, it can basically be concluded that CDDP is superior to ISMN in the treatment of elderly patients with UAP, and its effect is rapid and effective, although adverse reactions are mainly headache, redness, dizziness, and other symptoms; in a small amount of research nausea symptoms will appear. Therefore, CDDP is more suitable for the treatment of UAP in the elderly. However, our findings must be handled with care because of the small sample size and low quality of clinic trials cited. Other rigorous and large-scale RCTs are needed to confirm these results.

## Figures and Tables

**Table 1:** Characteristics of included studies.

Author, year	Cases (T/C)	Diagnostic standard	Age (years)	Sex	Dosage
			Range, mean	Male/female	T/C
Ren, 2013	32/23	DCWHO	T: 45~67, 56.2 C: 39~66, 54.8	23/9	T: 10 capsules/time, 3 times/d, oral C: 30 mg/time, 1 time/d, oral

Liu, 2005	33/32	DCWHO	T: 43~65, 52.3 ± 7.5 C: 41~64, 53.6 ± 8.4	T: 18/15 C: 19/13	T: 10 capsules/time, 3 times/d, oral C: 20 mg/time, 3 times/d, oral

Shi, 2013	40/40	DCWHO	60~83, 6.75 ± 6.32	43/37	T: 10 capsules/time, 3 times/d, oral C: 50 mg/time, 1 time/d, oral

Zhang, 2016	45/45	DCWHO	T: 55~83, 64.9 ± 6.2 C: 56~82, 67.3 ± 6.4	T: 30/15 C: 32/13	T: 10 capsules/time, 3 times/d, oral C: 10 mg/time, 3 times/d, oral

Zhang, 2014	80/80	NR	32~78,65.32 ± 3.10	87/73	T: 10 capsules/time, 3 times/d, oral C: NR

Jing, 2014	84/84	DCWHO	62~84,68.8 ± 5.48	88/80	T: 10 capsules/time, 3 times/d, oral C: 50 mg/time, 1 time/d, oral

Li, 2012	60/52	DCWHO	NR	NR	T: 10 capsules/time, 3 times/d, 5% glucose 250 ml, oral C: 10 mg/time, 3 times/d, 5% glucose 250 ml, oral

Li, 2016	43/42	NR	T: 61~84, 69.5 ± 5.6 C: 60~83, 69.3 ± 5.4	T: 26/17 C: 25/17	T: 10 capsules/time, 3 times/d, oral C: 50 mg/time, 1 time/d, oral

Yang, 2015	32/32	DCWHO	T: 62~84, 69.4 ± 5.8 C: 60~81, 68.2 ± 5.4	T: 18/14 C: 19/13	T: 3 tablets /time, 3 times/d, oral C: 50 mg/time, 1 time/d, oral

Wang, 2014	75/75	DCWHO	T: 60~82, 68.8 ± 6.5 C: 61~80, 66.7 ± 6.3	T: 40/35 C: 38/37	T: 10 capsules/time, 3 times/d, oral C: 50 mg/time, 1 time/d, oral

Sheng, 2015	40/40	NR	T: 58~80, 65.9 ± 3.6 C: 57~82, 66.8 ± 4.1	T: 17/23 C: 16/24	T: 10 capsules/time, 3 times/d, oral C: 10 mg/time, 3 times/d, oral

Su, 2015	106/106	NR	58~83, 67.3 ± 6.9	127/85	T: 10 capsules/time, 3 times/d, oral C: 50 mg/time, 1 time/d, oral

Shao, 2013	40/40	DCWHO	60~80, 67.75 ± 6.32	43/37	T: 10 capsules/time, 3 times/d, oral C: 10 mg/time, 3 times/d, oral

Guo, 2013	129/107	DCWHO	42~70, 51.9 ± 13.6	161/75	T: 10 capsules/time, 3 times/d, oral C: 20 mg, intravenous drip, 5% glucose 250 ml

Chen, 2009	46/46	ACC/AHA	T: 41~69, 48.5 ± 12.9 C: 45~72, 49.3 ± 11.1	T: 32/14 C: 31/15	T: 10 capsules/time, 3 times/d, oral C: 20 mg/time, 3 times/d, oral

Ma, 2012	70/70	CMACB	61~78, 67.1 ± 6.4	103/37	T: 10 capsules/time, 3 times/d, oral C: 30 mg/time, 2 times/d, oral

He, 2011	45/45	DCWHO	T: 66.4 C: 65.7	T: 25/20 C: 24/21	T: 10 capsules/time, 3 times/d, oral C: 30 mg/time, 1 time/d, oral

Zeng, 2014	50/50	DCWHO	60~76, 67.8 ± 6.5	66/34	T: 10 capsules/time, 3 times/d, oral C: 20 mg/time, 2 times/d, oral

Geng, 2015	63/63	DCWHO	44~75, 53.5 ± 7.6	65/61	T: 10 capsules/time, 3 times/d, oral C: NR

Ma, 2014	42/41	DCWHO	55~73, 62.5 ± 4.2	50/33	T: 10 capsules/time, 3 times/d, oral C: 30 mg/time, 1 time/d, oral

Gao, 2013	44/44	DCWHO	T: 52~76, 60.8 ± 7 C: 53~78, 61.5 ± 8	T: 27/17 C: 29/15	T: 10 capsules/time, 3 times/d, oral C: 10 mg/time, oral

**Table 2:** Clinical efficacy, ECG efficiency, and adverse reaction information.

Author, year	Treatment/week	Effective clinical efficacy		Efficacy of ECG		Adverse reaction rate		Outcome measures
		(Effective/total)		(Effective/total)		(Effective/total)		
		Treatment control		Treatment control		Treatment control		
Ren, 2013	4	29/32	17/23	28/32	16/32	NR		

Liu, 2005	4	29/33	24/32	25/33	19/32	NR		

Shi, 2013	4	38/40	32/40	NR		9/40	10/40	Blood viscosity

Zhang, 2016	4	42/45	27/45	NR		6/45	13/45	

Zhang, 2014	4	78/80	69/80	NR		NR		Blood viscosity

Jing, 2014	4	75/84	61/84	NR		9/84	22/84	

Li 2012	4	54/60	38/52	NR		NR		

Li, 2016	4	40/43	30/42	NR		5/43	5/42	Blood viscosity

Yang, 2015	4	29/32	22/32	NR		NR		Angina pectoris frequency and attack time

Wang, 2014	4	72/75	59/75	NR		16/75	17/75	Blood viscosity

Sheng, 2015	4	37/40	30/40	NR		NR		

Su, 2015	4	99/106	86/106	NR		14/106	11/106	Blood viscosity

Shao, 2013	4	38/40	30/40	NR		9/40	10/40	

Guo, 2013	4	117/129	81/107	118/129	94/129	NR		Cardiac function index (SV, CO, LEVF)

Chen, 2009	4	43/46	38/46	NR		2/46	2/46	Cardiac function index (SV, CO, LEVF)

Ma, 2012	4	65/70	53/70	64/70	51/70	NR		Angina pectoris frequency and attack time

He, 2011	8	43/45	39/45	NR		NR		Angina pectoris frequency and attack time

Zeng, 2014	8	46/50	34/50	NR		7/50	15/50	

Geng, 2015	8	57/63	47/63	59//63	45/63	5/63	4/63	

Ma, 2014	8	40/42	28/41	NR		NR		Angina pectoris frequency and attack time

Gao, 2013	8	41/44	37/44	NR		NR		Angina pectoris frequency and attack time

## Data Availability

The data used to support the findings of this study are available from the corresponding author upon request.
